# Reproductive Maturation of Meagre *Argyrosomus regius* (Asso, 1801) Reared in Floating Cages

**DOI:** 10.3390/ani13020223

**Published:** 2023-01-07

**Authors:** Rosa Zupa, Edmond Hala, Gianluca Ventriglia, Chrysovalentinos Pousis, Letizia Passantino, Angelo Quaranta, Aldo Corriero, Caterina De Virgilio

**Affiliations:** 1 Department of Veterinary Medicine, University of Bari Aldo Moro, 70010 Valenzano, Italy; 2 Department of Animal Production, Faculty of Agriculture and Environment, Agricultural University of Tirana, 1030 Tirana, Albania; 3 Department of Precision and Regenerative Medicine and Ionian Area, Section of Veterinary Clinics and Animal Production, University of Bari Aldo Moro, 70010 Valenzano, Italy; 4 Department of Biosciences, Biotechnologies and Environment, University of Bari Aldo Moro, 70124 Bari, Italy

**Keywords:** broodstock management, reproduction in captivity, fish oogenesis, fish spermatogenesis, fish reproduction

## Abstract

**Simple Summary:**

The meagre (scientific name: *Argyrosomus regius*) is a fish species inhabiting the Mediterranean Sea and the eastern Atlantic Ocean. Consumer appreciation and good market quotations induced the aquaculture industry to invest in its domestication. However, when reared in tanks, meagre males produce milt of low quality and quantity, and females do not finalise oogenesis and do not spawn spontaneously. The reproductive dysfunctions affecting meagre reared in tanks, can be alleviated through the use of hormone administration or through the association of hormone treatments with photo-thermal control. In this study, the reproductive maturation of meagre, reared in sea cages under routine farming condition, was assessed. It was observed that female and male meagre produced in captivity (hatchery), transferred as juveniles to sea cages located in the Gulf of Taranto (Italy), and reared until sexual maturity under routine commercial conditions, are able to produce and release gametes spontaneously. Meagre reproduction, under routine farming conditions, may represent an opportunity for the expansion of meagre EU aquaculture production; this actually relies on limited amounts of fingerlings, produced by few hatcheries.

**Abstract:**

The meagre *Argyrosomus regius* (Asso, 1801) is a promising aquaculture species that shows reproductive dysfunctions when reared in tanks. The aim of this study was to assess the capacity of meagre, reared in cages under routine farming conditions, to mature gonads and reproduce spontaneously. Meagre adults, reared in a fish farm located in the Gulf of Taranto (Italy), were sampled from March to July 2021. The gonadosomatic index and sex steroid plasma concentrations increased from March–April to June, and then decreased in July. In March–April, most of the females showed perinucleolar or cortical alveoli oocytes as the most advanced stages in the ovaries, and most of the males had testes at early spermatogenesis stage. In June, most of the sampled females had oocytes at late vitellogenesis or early post-vitellogenesis stages, and males had seminiferous tubules filled with spermatozoa. In July, most of the females had signs of previous spawning, and males showed scarce amounts of luminal spermatozoa. The present study demonstrated the capacity of meagre, reared in sea cages under commercial conditions, to carry out gametogenesis and spontaneously release gametes. Meagre reproduction, under routine farming conditions, may represent an opportunity for the expansion of meagre aquaculture production.

## 1. Introduction

The meagre *Argyrosomus regius* (Asso, 1801) is a promising niche species that is increasingly appreciated by consumers [1], and whose EU fishery and aquaculture production is undergoing a progressive growth [2]. The consumption of meagre products of fishery origin has grown to the point that, in some areas of the Mediterranean, wild fish stocks have been significantly depleted, and restocking activities have been implemented [3,4]. 

Different aspects of meagre aquaculture technology have been developed so far, including larval rearing [5,6,7,8,9,10], on-growing and nutrition [11,12,13], and product quality assessment [14,15]. Moreover, in the last decade, effective reproduction control protocols have been developed for broodstock reared in tanks. Thanks to these technologies, based on hormone administration [16,17], or hormone administration associated with thermal/photothermal control [18,19,20,21], large amounts of high quality eggs and sperm, as well as high fertilization and larval survival rates, have been obtained. 

The large-scale production of a fish species needs the achievement of reproduction control [22]. The state of confinement triggers the onset of reproductive dysfunctions [22,23,24,25] that prevent the release of fertile gametes for the development of larval breeding. Meagre broodstock, reared in tanks under controlled temperature and photoperiod regimes, showed reproductive dysfunctions of mild (males) or severe degree (females). The main reproductive dysfunction observed in meagre females, of both wild origin and born in captivity (F1 generation), was the failure of the oocytes to reach the final maturation, and their ability to ovulate and spawn [3,18,26,27]; meanwhile, meagre males were able to finalize gametogenesis and release sperm [18,26,28,29], but they produced milt of low quality [18,21,26] and of scarce volume [21,30,31]. Nevertheless, the administration of an agonist of the gonadotropin-releasing hormone (GnRHa), via sustained-release implants or multiple injections, has proven to be effective in inducing the release of good-quality fertile eggs and improving sperm quality [18,19,20,21,27,31,32,33]. 

Despite the increasing market demand, EU meagre aquaculture production relies on just a few hatcheries, which limits the potential development of a large-scale production of this species [16,34]. To date, there is scarce information regarding the reproduction of meagre reared in floating sea cages [3,35], since the fish are confined in cages only for ongrowing and marketing purposes [1]. The aim of this study was to analyse the reproductive maturation of adult meagre, reared in floating sea cages in the northern Gulf of Taranto (Ionian Sea, Italy), and assess their potential to mature gonads and reproduce spontaneously under routine commercial conditions. The spontaneous or induced reproduction of meagre, confined in floating cages, could offer the advantage of considerably increasing the production of fertilised eggs for the needs of the farming industry.

## 2. Materials and Methods

### 2.1. Ethical Statement

No authorization from the Ethical Committee was requested for the present study, because all the specimens used were destined for the market and were purchased immediately after routine commercial harvesting.

### 2.2. Sampling 

A total of 49 meagre (25 females and 24 males) were used for the present study (Table 1). Fish belonged to a stock purchased as fingerlings (15 g body mass) from a French hatchery in 2015 and reared for six years in a floating sea cage of 3500 m^3^ belonging to the fish farm Rehomare InMare S.r.l. (Gallipoli, Italy). The age of the sampled fish was beyond that of first sexual maturity of the Mediterranean wild meagre population [29,36]. Fish were reared at the density of approximately 23 kg m^3^ and fed daily with dry extruded feed (Biomar, EFICO 2152, 9 mm). 

Five fish samplings were carried in 2021, during the period March–July, which encompasses the reproductive season of this species in the Mediterranean [3,26,29,36]. The samplings were carried out on 25 March (five females and five males), 28 April (seven females and two males), 9 June (six females and four males), 9 July (three females and six males) and 14 July (four females and seven males). Based on the results of the gonad histological analysis (see Results section), the sampled fish were clustered in three groups: fish sampled in March–April (early gametogenesis period); fish sampled in early June (ripening period); and fish sampled in July (spawning/post-spawning period). 

From each fish, biometric data were recorded (total length, TL, in cm; total body mass, BM, in kg; gonad mass, GM, in g) (Table 1). Blood samples were taken from the caudal vasculature and one-centimetre-thick cross-sections were taken from the gonads and fixed in Bouin’s solution. Due to unexpected difficulties that occurred during the harvesting operations, it was not possible to collect blood samples from fish sampled on 9 July. The relative gonad mass, gonadosomatic index, was estimated as GSI = 100 × GM × BM^–1^.

### 2.3. Gonad Histological Analysis

For the histological analysis, fixed gonads were dehydrated in ethanol, clarified in xylene, and embedded in paraffin wax. Subsequently, four-μm-thick sections were stained with haematoxylin-eosin. 

The reproductive state of the females was assessed according to Corriero et al. (2003, 2007) [37,38], on the basis of the most advanced oocyte population, and the presence of post-ovulatory (POFs) and atretic vitellogenic follicles. The identification of atretic follicles followed the description of Hunter and Macewicz (1985) [39]. The initial atresia of vitellogenic follicles (α atresia) was characterized by the disintegration of the oocyte nucleus and cytoplasmic organelles, and by zona radiata fragmentation. The percentage of atretic vitellogenic follicles was calculated in meagre females whose ovaries showed oocytes in late vitellogenesis. Digital fields of ovary sections were photographed randomly using a digital camera (DFC 420; Leica Microsystems, Cambridge, UK) connected to a light microscope (DIAPLAN; Ernst Leitz GmbH, Wetzlar, Germany) and all the follicles in late vitellogenesis were counted. The percentage of atretic follicles was calculated as 100 × AF, where AF is the number of atretic follicles/total number of late vitellogenic follicles.

The reproductive state of males was assessed on the basis of the type of spermatogenic cysts and the amount of spermatozoa present in the lumen of seminiferous lobules, according Corriero et al. (2007) [38]. 

At least 50 seminiferous tubules were selected randomly from the testicular sections stained with haematoxylin-eosin. The seminiferous lobule diameter was measured on a digital field photographed with a 10× objective, using the same equipment used for the quantification of the follicular atresia. Measurements were performed using an image analysis software (Leica Application Suite, version 3.3.0; Cambridge, UK).

### 2.4. Sex-Steroid Plasma Level Measurement

Blood was centrifuged (5000 rpm for 10 minutes) and plasma was taken and stored at −20 °C until the analysis of 17β-estradiol (E_2_) (females) and 11-Ketotestosterone (11-KT) (males). Steroid analyses were performed using ELISA kits (Cayman Chemical Company, Ann Arbor, MI, USA), according to the manufacturer’s instructions. Undiluted plasma was used for E_2_; 1:20 and 1:70 plasma dilutions were used for 11-KT. Before running the ELISA tests, 250 μL of plasma were extracted twice with 2 mL diethyl ether by vigorous vortexing (Gyromixer 300–100, TKA, Milan, Italy) for 3 min, and then frozen for 30 min at −63 °C. Finally, the supernatant organic phase was collected and evaporated under a stream of nitrogen. Before running the ELISA test, samples were reconstituted in a reaction buffer.

### 2.5. Statistical Analysis

The normal distribution of data was assessed through the Shapiro–Wilk W test. Differences in GSI and sex steroid plasma concentrations in consecutive phases of the reproductive cycle were assessed by an ANOVA, followed by Duncan’s new multiple range post hoc test. Differences in the diameter of seminiferous tubules in consecutive phases of the reproductive cycle were assessed by a Mann–Whitney U test. Statistical analyses were performed using MS Office Excel 365, and statistical significance was accepted for *p* < 0.05. Results were expressed as mean ± SD. 

## 3. Results 

### 3.1. Gonadosomatic Index and Sex Steroid Plasma Concentrations

**Figure 1:**
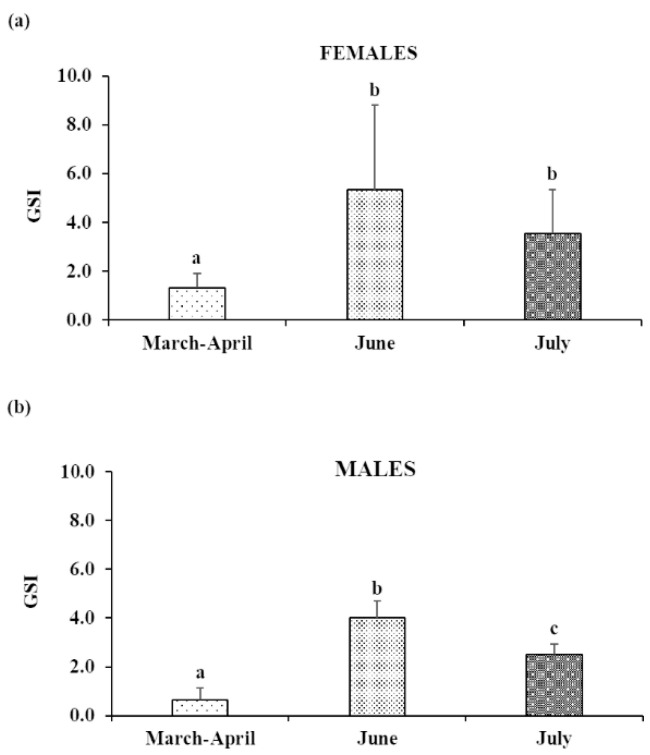
Mean (± SD) gonadosomatic index (GSI) of female (**a**) and male (**b**) meagre reared in floating sea cages in the Gulf of Taranto (North Ionian Sea, Gallipoli, Italy). Different letters indicate statistically significant differences between consecutive periods (ANOVA; *p* < 0.05).

In both sexes, GSI increased significantly from March–April to June and then decreased in July, although the latter GSI change was statistically significant only in males (Figure 1). 

**Figure 2:**
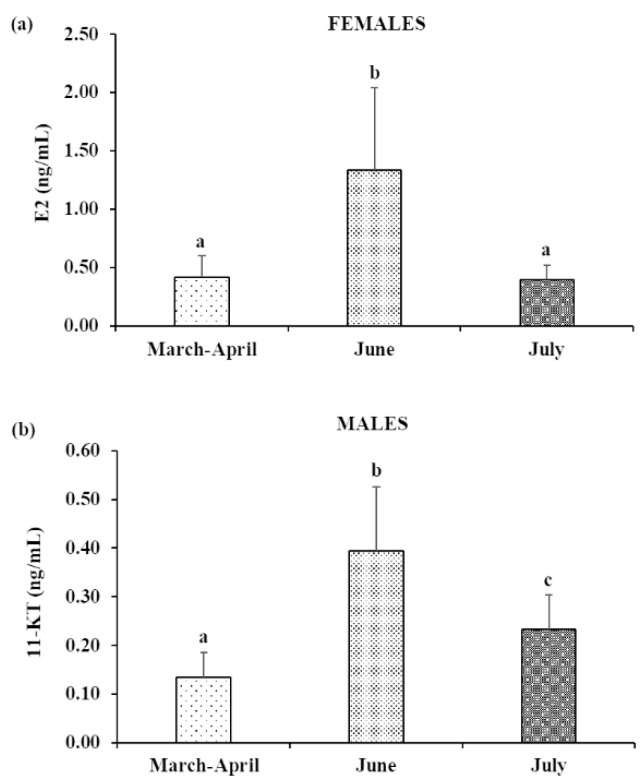
Mean (±SD) plasma (**a**) 17-β estradiol (E_2_) (femalesand (**b**) 11-Ketotestosterone (11-KT) (males) concentrations in meagre reared in floating sea cages. Different letters indicate statistically significant differences between consecutive periods (ANOVA; *p* < 0.05).

Plasma concentrations of E_2_ and 11-KT showed the same trend as GSI. In fact, both the hormone plasma levels showed a significant increase from March–April to June, followed by a significant decrease in July (Figure 2). 

### 3.2. Histological Analysis of Ovaries 

**Figure 3:**
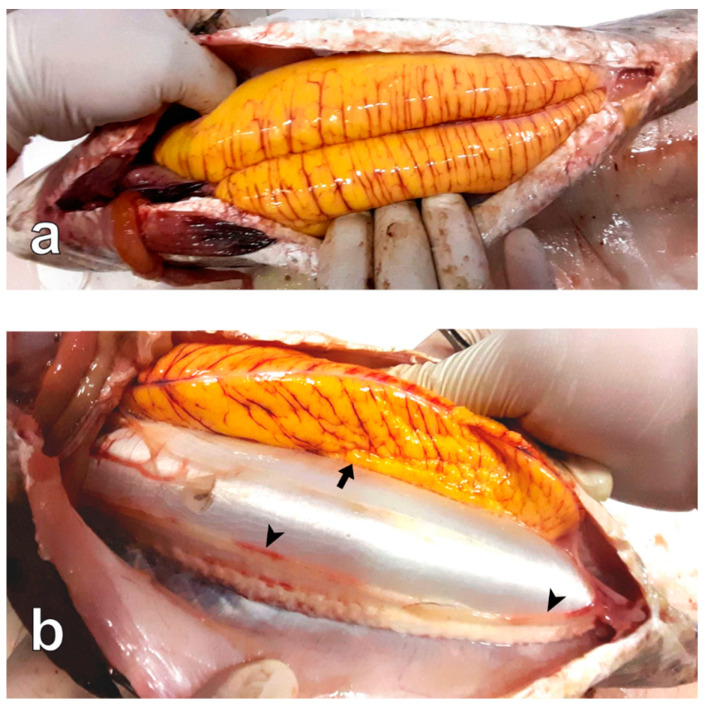
Meagre females sampled on 9 July 2021. (**a**) Specimen showing well developed ovaries. (**b**) Specimen showing a well-developed ovary (arrow) and an undeveloped ovary (arrowhead).

Some individuals showing the two ovaries in markedly different development states were found during all the reproductive phases (36% of the sampled females) (Figure 3). From these fish, only the most developed ovary was analysed. 

**Figure 4:**
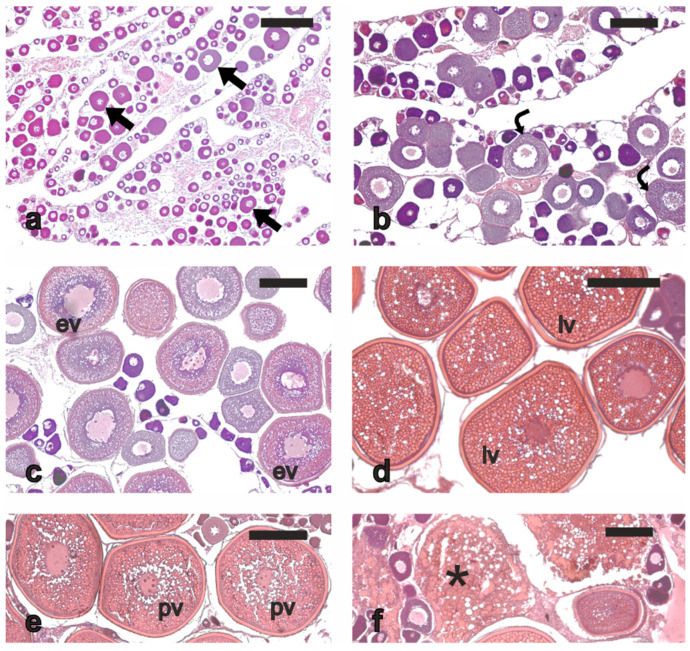
Micrographs of ovary sections from cage-reared meagre in different reproductive conditions. (**a**) Ovary from a quiescent fish sampled in April showing the perinucleolar stage as most advanced oocyte stage. (**b**) Oocytes at cortical alveoli stage in the ovary of a specimen sampled in April. (**c**) Early vitellogenic oocytes in the ovary of a fish sampled in April. (**d**) Late vitellogenic oocytes in the ovary of a meagre sampled in June. (**e**) Early post-vitellogenic oocytes, characterised by initial coalescence of yolk platelets and lipid droplets, in the ovary of a fish sampled in June. (**f**) Atretic vitellogenic follicles in the ovary of a fish sampled in June. Haematoxylin-eosin staining. Magnification bars: 250 μm. Arrow, perinucleolar stage oocyte; asterisk, α atretic oocyte; curved arrow, oocyte at cortical alveoli stage; ev, early vitellogenic oocyte; lv, late vitellogenic oocyte; pv, post vitellogenic oocyte.

**Figure 5:**
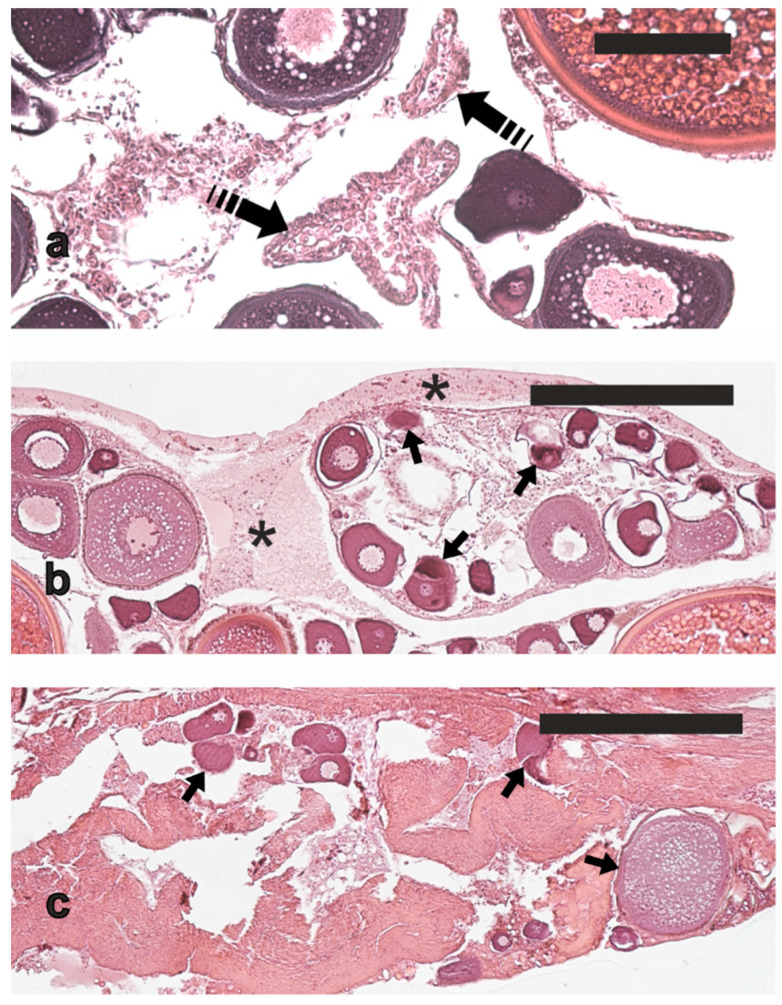
(**a**) Micrographs of ovary sections from a specimen sampled in July showing post-ovulatory follicles (POFs), a sign of recent spawning. (**b**,**c**) Micrographs of ovary sections from fish sampled in July showing a reduction in oocyte density, a plasma transudate rich in leucocytes (dark spots in the transudate) and a degeneration of pre-vitellogenic follicles. Haematoxylin-eosin staining. Magnification bars: 100 μm in (**a**) and 300 μm in (**b**,**c**). Arrow, degenerating pre-vitellogenic follicles; asterisk, plasma transudate; dashed arrow, post-ovulatory follicle.

In March-April, most of the females (*n* = 9) showed perinucleolar oocytes (Figure 4a) or cortical alveoli oocytes (Figure 4b) as their most advanced stages in the ovaries; three females had already entered the secondary oocyte growth, showing few early vitellogenic oocytes (Figure 4c). In June, most of the sampled females had ovaries with oocytes at late vitellogenesis or early post-vitellogenesis stages (maximum oocyte diameter 520–575 μm) (Figure 4d, e), as well as minor α atresia (<50% of α atretic vitellogenic oocytes) (Figure 4f); two females in this period were in a less advanced reproductive maturation state because they had cortical alveoli or early vitellogenesis as their most advanced oocyte stages. In July, five females had oocyte at the late vitellogenesis stage or early post-vitellogenesis as their most advanced oocyte stages (maximum oocyte diameter 450–590 μm), whereas two fish were in a less advanced stage of reproductive maturation, showing oocytes at early vitellogenesis as their most advanced oocyte stage. Among the five vitellogenic/post-vitellogenic females, three also had evident POFs (Figure 5a), the sign of very recent spawning (<48 h), and two showed a reduction in oocyte density, an increase in somatic tissue, a plasma transudate rich in leucocytes and a non-atretic degeneration of pre-vitellogenic follicles (Figure 5b, c), likely signs of less recent spawning (>48 h). All these vitellogenic females displayed minor α atresia of vitellogenic follicles (<50% of α atretic vitellogenic oocytes). 

**Figure 6:**
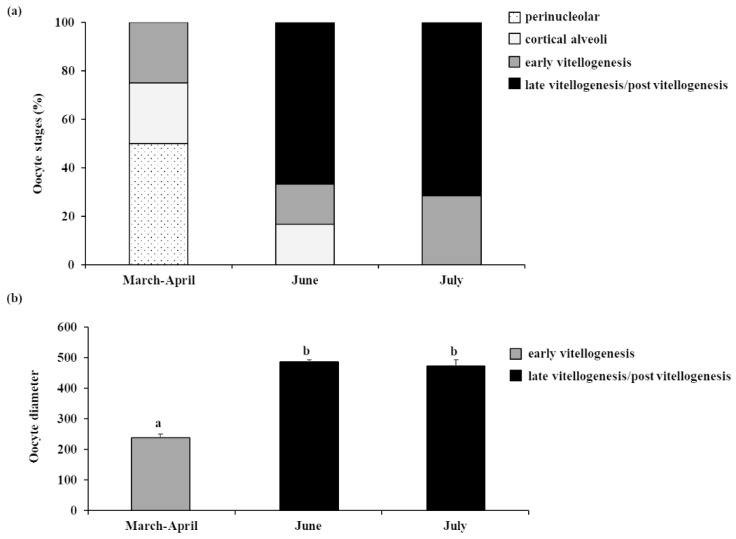
(**a**) Percentage of the different oocyte development stages and (**b**) mean diameter of the most advanced oocyte populations, observed in the ovaries of female meagre reared in floating sea cages. Different letters indicate statistically significant differences between consecutive periods (ANOVA; *p* < 0.05).

The percentage of oocytes in the different developmental stages and the trend of mean diameter of the most advanced oocyte populations are shown in Figure 6. 

### 3.3. Histological Analysis of Testes

**Figure 7:**
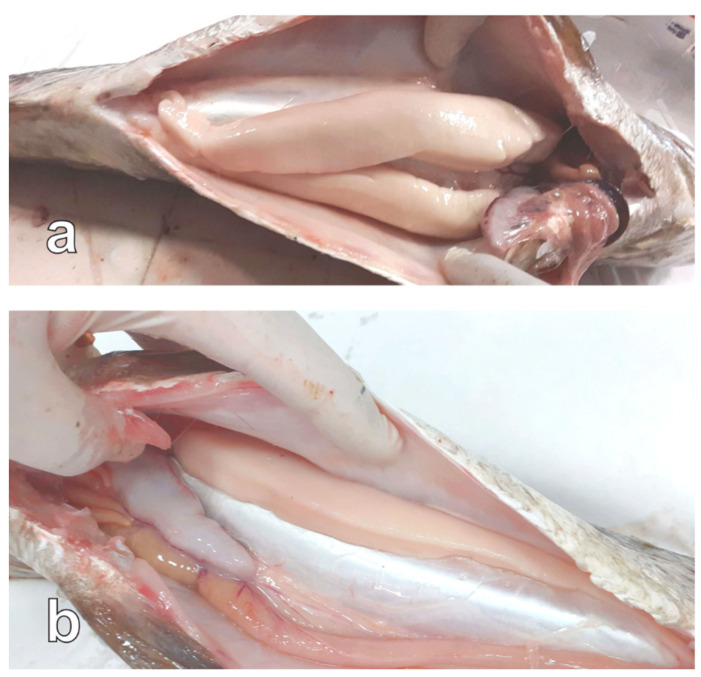
Meagre males sampled on 25 March 2021. (**a**) Specimen showing well developed testes. (**b**) Specimen showing only a well-developed testis.

Some individuals showing testes in markedly different development states were found during all the reproductive phases (29% of the sampled males) (Figure 7). From these fish, only the most developed testis was analysed. 

**Figure 8:**
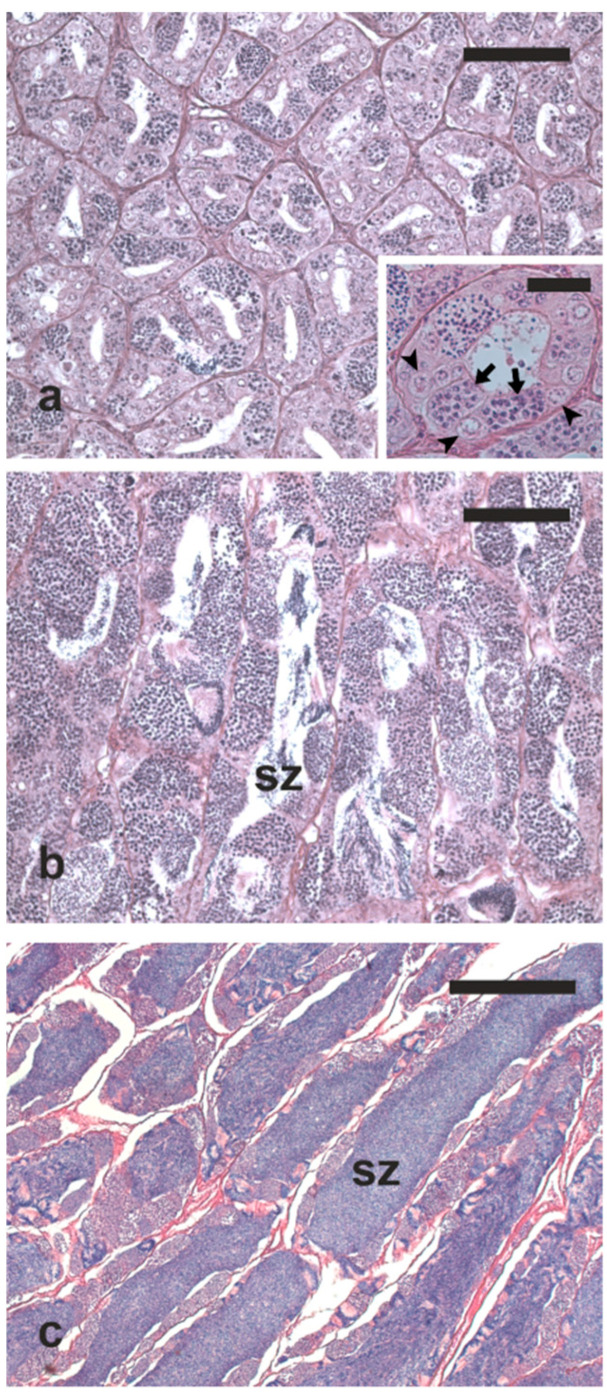
Micrographs of testis sections from cage-reared meagre in different reproductive conditions. (**a**) Testis in quiescent state sampled in March, whose germinal epithelium was mainly constituted by single spermatogonia and spermatogonial cysts (inset). (**b**) Testis in active spermatogenesis sampled in April, showing all stages of spermatogenesis and a limited number of luminal spermatozoa. (**c**) Mature testis sampled in June showing seminiferous tubules filled with spermatozoa. Haematoxylin-eosin staining. Magnification bars = 100 μm in (**a**,**b**); 30 μm in inset of (**a**); 300 μm in (**c**). Arrow, spermatogonial cyst; arrowhead, single spermatogonium; sz, spermatozoa.

Among the fish sampled in March-April, two had quiescent testes mainly showing single undifferentiated type A spermatogonia and few spermatogonial cysts (cysts containing type A or type B spermatogonia) (Figure 8a); the other fish had testes in active spermatogenesis showing all the germ cell types in the germinal epithelium and spermatozoa in the lumen of the seminiferous lobules (Figure 8b). The mean diameter of the seminiferous tubules of fish samples in this period was 92.4 ± 28.2 μm. 

In June, all the males were mature since they had testes with a thin germinal epithelium containing residual spermatogenetic activity and plenty of luminal spermatozoa (Figure 8c). The testes of fish sampled in this period had significantly larger seminiferous tubules (203.2 ± 23.8 μm), compared with those sampled in the previous period. One of the fish sampled in this period was fluent (spontaneously released sperm), and the other three released sperm upon slight compression of the abdominal wall. 

In July, one male was mature showing a thin germinal epithelium containing residual spermatogenic activity and plenty of luminal spermatozoa (this fish released sperm after abdominal pressure). The other sampled fish, despite still being in active spermatogenesis with germ cells in all the spermatogenesis stages, had scarce amounts of spermatozoa in the lumen of the seminiferous lobules. A significant decrease in the diameter of the seminiferous lobules (156.0 ± 22.4 μm) was observed in males during the spawning phase. 

## 4. Discussion

In the Mediterranean, tank-reared meagre broodstocks have been reported to fail to mature eggs and spawn spontaneously [3,26]. Spontaneous spawning of meagre reared in indoor tanks in the Mediterranean has been reported, as occasional event, by Mylonas et al. (2013) [26]. In fact, the two females that were reported to spawn spontaneously belonged to a broodstock that was used in spawning induction trials during the previous reproductive season [19]; according to the authors of the same study, “…*oocyte maturation is sporadic and inconsistent, and reliable spawning could be obtained using only exogenous hormones*”. Conversely, hatchery-produced meagre produced from parents caught from the wild in the eastern Atlantic and hormonally-induced to spawn, were reported to mature and spawn spontaneously when reared in indoor tanks under controlled photothermal conditions [27]. The different capacity of species to adapt to captivity and reproduce spontaneously between Mediterranean and Atlantic populations has been also reported for the greater amberjack *Seriola dumerili* and this was hypothesised to be due to genetic and/or environmental factors (for a review of greater amberjack reproduction, see Corriero et al., 2021 [40]).

The capacity of fish reared in sea cages to finalise gametogenesis and spawn spontaneously in captivity has occasionally been reported. Barramundi *Lates calcarifer* reared in floating net cages in the tropical northern Pacific Ocean, were reported to mature spontaneously and spawn large numbers of fertilised eggs over a prolonged spawning season [41]. In the framework of domestication experiments, Atlantic bluefin tuna *Thunnus thynnus* farmed in sea cages in the Mediterranean were found to have a limited capacity to finalise oogenesis and spawn spontaneously; however, they were highly responsive to the hormonal induction of spawning [38,42,43,44]. The capacity of Atlantic bluefin tuna to spawn spontaneously under routine farming conditions in the central Adriatic Sea has also been reported [45]. Moreover, a limited capacity to spawn spontaneously has been reported for greater amberjack caught from the wild as juveniles and reared in sea cages until sexual maturity [17].

In the present study, hatchery-produced meagre, bought as juveniles and commercially reared for six years in a floating sea cages located in the Gulf of Taranto, 1.2 m off the Apulian coast, showed the capacity to carry out gonad maturation according to a temporal schedule similar to that described for the wild Mediterranean population [36], as well as for tank-reared broodstocks [26], although with a slight temporal shift. In floating sea cages, meagre gametogenesis started in March or April, with some asynchrony among individuals. In early June, most of the females (2/3) had fully vitellogenic oocytes, and all the sampled males had mature testes and released sperm spontaneously or when the abdominal wall was slightly compressed. In July, clear histological signs of spawning were observed in the ovaries of the sampled females. In particular, post-ovulatory follicles were found in all the three females sampled in early July (9 July) which indicates that these females had released eggs during the two previous days. No post-ovulatory follicles were found in any of the four females sampled in mid-July (14 July); however, half of them showed regressing ovaries, whose characteristics (plasma transudate rich in leucocytes and the non-atretic degeneration of pre-vitellogenic follicles) suggest that they had spawned a few days before sampling. In males, the reductions observed in July in GSI, the seminiferous tubule diameter and the amount of luminal spermatozoa, are compatible with spontaneous sperm release in June/early July. The observed timing of gonad maturation and spawning was slightly delayed compared with both the wild meagre population in the Mediterranean [36] and the hatchery-produced meagre broodstock reared in tanks in Crete [26], which showed a reproduction peak in May–early June. The slight shift in the maturation/spawning period can be either due to the delay in spring sea surface temperature rise of the the Gulf of Taranto compared with more eastern Mediterranean locations, or simply due to normal yearly fluctuations in the reproductive cycle. Unfortunately, no data are available on the reproductive maturation of wild meagre populations from the same area of the present study. 

Sex steroid plasma concentrations observed in cage-reared meagre were in the range of those reported for tank-reared meagre and confirm previous observations that low steroid concentrations are enough to support the gametogenesis process in meagre [19,26]. 

The biometric, histological and endocrine data provided in the present study testify the ability of hatchery-produced meagre to start gametogenesis according to the normal temporal schedule of the wild population, to carry out normal vitellogenesis and spermatogenesis and—at least to some extent—to finalise gametogenesis and spawn in sea cages. Moreover, most of the females sampled in June and July showed fully vitellogenic oocytes whose maximum diameter, considering the shrinkage occurring in chemically-fixed and paraffin-embedded tissues [46,47], exceeded 600 μm. According to Mylonas et al. (2013) [19], meagre females are responsive to GnRHa treatments for spawning induction if they contain oocytes in full vitellogenesis with a diameter of 550 μm and very little atresia; meanwhile, males are responsive if they are in full spermiation, releasing sperm after compression of the abdominal wall. In the present study, most of the females and all of the males sampled in June and July fulfilled Mylonas et al.’s (2013) [19] conditions to be eligible for the hormonal induction of spawning. Moreover, the present study suggests that meagre may be capable of spawning spontaneously when reared in cages under suitable environmental and rearing conditions. The spontaneous reproduction of meagre reared in sea cages under routine commercial conditions deserves further investigation because it would reduce the industrial costs for broodstock management. Moreover, the number of cage-reared breeders, and then the amount of fertilised eggs, could be increased according to the production targets; meanwhile, the need for hormonal therapies, whose use in commercial fish farming is restricted by national and EU regulations, could be reduced. 

In the present study, no attempt was made to collect, count and analyse the quality of the released eggs, which would have been a difficult task due to the wide size of the rearing cages (3500 m^3^) and the fact that the cages are exposed to currents with an intensity up to 2.5 m s^–1^ (personal communication of the farm crew chief). To this aim, in future studies, smaller dedicated sea cages could be used to confine the broodstock, avoid repeated stressful harvesting, and limit egg dispersion through the application of egg collector devices. 

Interestingly, about one third of the analysed fish showed only one well-developed gonad, an anomaly that has never been reported in previous studies on meagre reproduction. The occurrence of unilateral gonad atrophy has already been reported [48,49,50]. In some cases, this finding was associated with environmental pollution [48,49]. The fish farm of the present study is located in an oligotrophic marine area, far from any river discharge, and is not affected by any source of industrial pollution; therefore, the effect of environmental pollution on the observed asynchrony in gonad development is implausible. It cannot be excluded that the repeated exposure to acute stress due to the routine commercial harvesting in the sea cage may have affected, in some way, the normal gonad development.

## 5. Conclusions

The present study demonstrated the capacity of meagre reared in sea cages under routine commercial conditions to carry out gametogenesis. The process of gametogenesis in sea cages did not seem to be significantly altered by the confinement in captivity: males reached full maturity and released sperm spontaneously or after gentle abdominal pressure, and females were able to finalise vitellogenesis and, at least to some extent, mature and release eggs. Further investigations are needed to verify whether oocyte maturation and egg releases were occasional events or if they occur every year on a regular basis, and to assess the quantity and quality of the spawning. If natural spawning will not prove to be reliable and repeatable, spawning will likely be achieved through hormonal induction; this is because all the males and most of the analysed females were potentially responsive to the commonly applied treatments. The production of meagre fertilised eggs under routine commercial conditions may represent an opportunity for the expansion of meagre aquaculture production. 

## Figures and Tables

**Table 1:** Biometric data of meagre reared in floating sea cages in the Gulf of Taranto (North Ionian Sea).

Sampling Period (Temperature)	Sex	Total Length (cm)		Total Body Mass (kg)		Gonad Mass (g)	
		mean ± SD	min–max	mean ± SD	min–max	mean ± SD	min–max
March–April 14–17 °C	f	69.5 ± 3.1	64.5–73.0	3.3 ± 0.3	2.5–3.8	43.6 ± 21.9	14.7–88.6
	m	70.7 ± 3.1	67.0–75.0	3.3 ± 0.3	2.9–3.7	21.6 ± 17.5	4.5–51.1
June 20–23 °C	f	71.9 ± 3.6	68.0–77.0	3.3 ± 0.6	2.6–3.9	189.2 ± 144.0	26.0–408.0
	m	72.4 ± 2.1	70.0–75.0	3.5 ± 0.4	3.2–4.0	142.3 ± 35.0	94.0–177.0
July 24–25 °C	f	71.4 ± 7.3	61.0–80.0	3.0 ± 0.5	2.3–3.9	102.4 ± 48.4	46.0–166.0
	m	69.6 ± 6.3	57.0–76.0	2.9 ± 0.6	1.5–3.5	71.2 ± 19.6	34.0–103.0

## Data Availability

Data will be available upon request to the corresponding author.
